# Diagnostic Value of Bronchoscopy in Detecting Laryngopharyngeal Disorders: Clinical Utility and Limitations

**DOI:** 10.3390/medicina61091617

**Published:** 2025-09-07

**Authors:** Deok Hyong Kim, Bo Hyoung Kang, Soo-Jung Um, Insu Kim

**Affiliations:** 1 Department of Internal Medicine, Dong-A University Hospital, Busan 49201, Republic of Korea; deokhyung@naver.com; 2 Department of Internal Medicine, Dong-A University College of Medicine, Busan 49021, Republic of Korea; eeyoun@dau.ac.kr (B.H.K.); sjum@dau.ac.kr (S.-J.U.)

**Keywords:** bronchoscopy, laryngeal diseases, laryngoscopy, malignancy, upper respiratory tract diseases

## Abstract

*Background and Objectives*: Flexible bronchoscopy is widely used for evaluating intrathoracic airway and pulmonary diseases. However, its diagnostic value in upper airway abnormalities, including those involving the larynx, pharynx, and proximal trachea, remains underexplored. We evaluated the diagnostic significance and effectiveness of bronchoscopy in assessing upper airway diseases, including those involving the larynx, based on real-world data. *Materials and Methods*: We conducted a retrospective observational study involving 2229 patients who underwent bronchoscopy between March 2019 and December 2023. Patients with abnormal upper airway findings during bronchoscopy were referred for further otolaryngological evaluation. Patients were categorized according to the experience of their bronchoscopist (with ≥100 procedures defining experienced). Abnormal findings were analyzed according to anatomical region (oral cavity, larynx, and vocal cords), disease status (benign vs. malignant), and patient demographics. Multivariate logistic regression was used to identify predictors of abnormal findings. *Results*: Among 2229 patients (mean age 65.4 years), 72 (3.2%) exhibited visible upper airway abnormalities. No significant differences were observed in the abnormality detection rates between experienced and inexperienced operators across all anatomical regions. However, the presence of malignant disease was significantly associated with a higher likelihood of detecting abnormalities (5.4% vs. 1.9%, *p* < 0.001). Multivariate analysis revealed that male sex (odds ratio [OR] 2.069, *p* = 0.017), age < 74 years (OR 2.404, *p* = 0.009), and malignancy (OR 3.030, *p* < 0.001) were independent predictors of abnormal findings. *Conclusions*: Flexible bronchoscopy can incidentally detect upper airway abnormalities, particularly in patients with malignancy, male sex, or younger age. These findings suggest that systematic inspection of the upper airway during bronchoscopy may offer additional diagnostic value, regardless of the operator’s experience. The integration of upper airway assessment into routine bronchoscopic practice may enhance the early detection of clinically significant lesions and improve comprehensive patient care.

## 1. Introduction

Flexible bronchoscopy is an essential and widely adopted diagnostic and treatment modality in respiratory medicine since its development in the 1960s, primarily intended to visualize and evaluate intrathoracic airways and parenchymal lung diseases, including lung malignancy [1]. It enables direct inspection of the tracheobronchial tree, targeted biopsy of lesions, bronchoalveolar lavage, and airway sampling, thus playing a central role in the diagnosis and management of various pulmonary conditions [2]. Despite its broad application in thoracic disease, however, the diagnostic potential of flexible bronchoscopy for assessing upper airway abnormalities—including lesions involving the oral cavity, oropharynx, and larynx—remains relatively underexplored.

Diseases involving the upper respiratory tract, particularly those of otolaryngological origin, may present subtle or non-specific symptoms such as hoarseness, dysphagia, chronic cough, or dyspnea [3,4]. These symptoms are frequently misdiagnosed as lower respiratory tract causes, particularly in patients with overlapping lung disease such as lung cancer, which can result in underdiagnosis or delayed recognition of laryngopharyngeal disease [5]. Delayed or missed diagnosis may lead to airway compromise, impaired phonation, and increased morbidity [6]. Traditional diagnostic methods, such as laryngoscopy, provide excellent visualization of the vocal cords and supraglottic structures; however, their ability to assess subglottic and proximal tracheal abnormalities is limited [7,8,9]. Imaging modalities such as computed tomography (CT) and magnetic resonance imaging (MRI) offer detailed anatomical assessment; however, they may miss mucosal surface lesions or dynamic airway changes [8,10].

Therefore, this study aimed to evaluate the diagnostic significance and effectiveness of bronchoscopy in assessing upper airway diseases, including those involving the larynx, based on real-world data. 

## 2. Materials and Methods

### 2.1. Study Population

We conducted a retrospective observational study using data collected from patients who underwent bronchoscopy between March 2019 and December 2023 according to the Strengthening the Reporting of Observational Studies in Epidemiology statement. Patients with abnormal findings in the oropharynx after bronchoscopy underwent further laryngoscopic evaluation at the Department of Otolaryngology.

This retrospective study was conducted in accordance with the Declaration of Helsinki and approved by the Institutional Review Board of Dong-A University Hospital (Protocol# DAUHIRB-24-032). The requirement for written informed consent was waived owing to the retrospective design of the study.

### 2.2. Data Collection

Baseline characteristics included age, sex, presumptive diagnosis, bronchoscopic findings, presence or absence of abnormalities and details, otolaryngology (OL) consultation status or results, final diagnosis, and the presence of malignant or benign disease. Only cases with histologically confirmed malignant disease were included.

### 2.3. Bronchoscopy Operator

Bronchoscopy outcomes can vary significantly depending on the operator, highlighting the need for a learning curve to acquire a certain level of skill while performing bronchoscopy. Voduc N et al. reported that bronchoscopy skill acquisition differs among trainees, with a notable variation in the number of procedures required to reach competency between high- and low-performing groups [11]. Specifically, the high-performing group required only 25 procedures, whereas the low-performing group needed approximately 100. Furthermore, Mohan A et al. recommended performing at least 100 bronchoscopies (including 50 supervised and 50 independently performed) to ensure basic procedural competency [12]. Based on these findings, the current study defined the threshold between experienced and inexperienced bronchoscopists as 100 procedures [13].

### 2.4. Additional Consultation

The definition of abnormal findings included mucosal lesions and morphological structures not normally observed in the typical anatomical configuration, anatomical deformities, and additional masses, as well as depigmentation and discoloration identified via visual inspections. If abnormal findings were observed in the laryngopharyngeal or oral cavity during bronchoscopy, the patient was referred to the Department of Otolaryngology for further evaluation. In most cases, the Department of Otolaryngology conducted additional assessments; however, such evaluations could not always be performed due to scheduling conflicts or patient refusal.

### 2.5. Statistical Analysis

Variables were expressed as numbers with percentages for categorical data and as means with standard deviations for continuous data, as appropriate. To compare the two independent groups, the chi-squared test or Fisher’s exact test was used for categorical variables, whereas the independent *t*-test was used for continuous variables, depending on the normality of the distribution. Multivariate logistic regression analysis was conducted to identify clinical factors associated with laryngeal and vocal cord abnormalities. The odds ratios (ORs) and 95% confidence intervals (CIs) were calculated using logistic regression models. All statistical analyses were performed using Statistical Package for the Social Sciences (SPSS) software (version 22.0; SPSS Inc., Chicago, IL, USA). Statistical significance was set at *p* < 0.05.

## 3. Results

### 3.1. Patients

**Figure 1:**
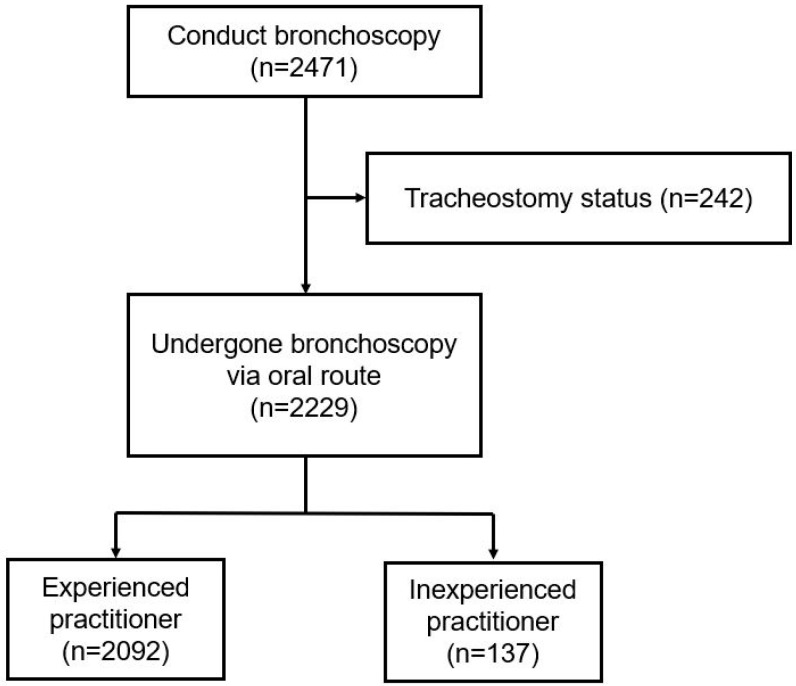
Flow diagram of study subjects.

In total, 2471 patients underwent bronchoscopy during the study period. However, 242 patients who underwent tracheostomy were excluded from the study, leaving 2229 patients (Figure 1).

The mean age of the included group was 65.4 years, and 1413 men accounted for 63.4% of the group. Bronchoscopy was performed by experienced practitioners in 93.9% of cases (2092 cases) and by inexperienced practitioners in 6.1% of cases (137 cases). Of the patients, 869 (39%) had malignant diseases and 72 (3.2%) had abnormal findings after bronchoscopy. Of these, 40 (56%) underwent additional consultations with an otolaryngologist (Table 1).

### 3.2. Bronchoscopy Operator

The detection of abnormal findings in each anatomical region was analyzed based on whether an experienced or inexperienced operator performed the procedures. In total, 2092 and 137 procedures were performed by experienced operators and inexperienced operators, respectively. These findings were categorized according to anatomical regions, namely, the oral cavity, larynx, and vocal cords (Table 2). In our cohort, no abnormal findings were observed in the oropharynx, although this region was systematically inspected during bronchoscopy.

In the oral cavity region, no abnormalities were observed in 137 procedures performed by inexperienced practitioners. Among the 2092 procedures performed by the experienced practitioners, only one abnormality was identified. No statistically significant difference was observed between the two groups (*p* = 1.000). In the laryngeal region, 137 bronchoscopies were performed by inexperienced practitioners, and none of them revealed any abnormalities. In contrast, among the 2092 bronchoscopies performed by experienced practitioners, abnormalities were detected in 21 cases (1%); however, no statistically significant difference was observed between the two groups (*p* = 0.636). Abnormalities in the vocal cord region were detected in 3.6% (5/137) of the cases examined by inexperienced practitioners and in 2.2% (47/2092) of those examined by experienced practitioners; however, the difference between the two groups was not statistically significant (*p* = 0.248). In the oral cavity and larynx, abnormal findings were observed in 5 and 68 patients in the inexperienced and experienced operator groups, respectively; however, the difference was not statistically significant (*p* = 0.802).

### 3.3. Presence of Malignancy

Of all patients, 1360 (61%) and 869 had benign and malignant diseases, respectively. In the oral cavity, no abnormalities were observed in patients with benign diseases, whereas one abnormality was identified in patients with malignant diseases; however, this difference was not statistically significant (*p* = 0.390). In the laryngeal area, 6 and 15 abnormal findings were detected in patients with benign and malignant diseases, respectively. A statistically significant difference was observed between the two groups (*p* = 0.002). In the vocal cord area, 22 and 30 abnormalities were observed in patients with benign and malignant diseases, respectively. A statistically significant difference was observed between the two groups (*p* = 0.005). When analyzed across the entire group, 26 (2%) and 47 (5%) abnormal findings were detected in patients with benign and malignant disease, respectively. A statistically significant difference was observed between patients with malignant and benign diseases regarding the detection of abnormal bronchoscopic findings (*p* = 0.000) (Table 3).

### 3.4. Multivariate Analysis for the Abnormal Finding Group

Multivariate logistic regression analysis was performed to identify factors associated with the presence of significant abnormalities. The results demonstrated that male sex was significantly associated with a higher likelihood of detecting abnormal findings, with an OR of 2.069 (95% CI, 1.138–3.761; *p* = 0.017), indicating that male patients were more than twice as likely to exhibit abnormalities as female patients. Additionally, younger age (<74 years) was identified as a significant factor, with patients under 74 years having an OR of 2.404 (95% CI, 1.247–4.634; *p* = 0.009) compared to those aged ≥74 years, suggesting a higher prevalence of abnormalities in the younger group. The presence of malignant disease was strongly associated with abnormal findings, with an OR of 3.030 (95% CI, 1.829–5.017; *p* < 0.001), indicating that patients with malignancy were three times more likely to present with abnormalities than those with benign disease. However, the operator experience was not significantly associated with abnormality detection. The OR for procedures performed by experienced practitioners was 0.648 (95% CI, 0.251–1.672; *p* = 0.370), suggesting no statistically significant difference in diagnostic performance between experienced and inexperienced examiners (Table 4).

## 4. Discussion

This study aimed to evaluate the diagnostic utility of flexible bronchoscopy in detecting upper airway abnormalities, particularly those involving the oral cavity, larynx, and vocal cords, using real-world data from a large cohort of patients who underwent bronchoscopy over a nearly five-year period.

Bronchoscopy plays a critical role in evaluating upper airway diseases by enabling direct visualization of the larynx, trachea, and proximal bronchi [1,2,12]. It allows clinicians to identify structural abnormalities, mucosal lesions, dynamic airway collapse, and foreign bodies that may not be evident in imaging studies [12,14]. In addition to diagnostic inspection, bronchoscopy facilitates targeted sampling through biopsy or bronchoalveolar lavage, providing histopathologic or microbiologic confirmation [13,15]. Its real-time assessment capability is particularly valuable in cases of unexplained hoarseness, stridor, chronic cough, or suspected upper airway obstruction [16]. Overall, bronchoscopy offers a safe, minimally invasive, and highly informative approach in the diagnostic workup of upper airway conditions [1,12]. Flexible bronchoscopy has been widely adopted as a diagnostic and therapeutic tool for lower respiratory tract diseases, including lung cancer; however, its role in assessing upper airway pathology remains under-recognized and under-investigated. Our findings highlight the diagnostic significance of incidental upper airway findings during bronchoscopy, along with several key clinical implications.

We observed that operator experience was not significantly associated with the detection of abnormalities in the oral cavity, larynx, or vocal cords. Experienced practitioners performed the majority of the bronchoscopic procedures in our cohort (93.9%); however, the detection rates of upper airway abnormalities were not significantly different from those of procedures performed by inexperienced operators. This finding may appear counterintuitive given the assumption that procedural skill influences diagnostic yield; however, the finding is likely attributable to the anatomical exposure inherent in the procedure. We observed that even relatively inexperienced operators may adequately detect gross mucosal lesions, masses, or asymmetries without requiring advanced navigational skills because the upper airway, including the oropharynx and laryngeal inlet, is visualized early in the bronchoscopic examination. This result is consistent with the concept that, while procedural experience is essential for navigating distal airways and performing complex interventions, such as transbronchial biopsies or endobronchial ultrasound-guided transbronchial needle aspiration, the detection of proximal upper airway pathology may rely more on thorough visual inspection and clinical suspicion than on procedural volume alone.

Furthermore, the presence of an underlying malignancy was significantly associated with a higher rate of abnormal findings in the upper airway, particularly in the larynx and vocal cords. Among patients with malignant diseases, abnormal findings were three times more likely to be observed than in those with benign conditions, as confirmed by multivariable logistic regression. This observation aligns with previous reports indicating that head and neck malignancies, including primary lung cancers, may involve the upper aerodigestive tract through either direct extension, metastasis, or synchronous tumors [17,18,19]. Additionally, airway inflammation or irritation secondary to tumor burden or treatment (including radiation or chemotherapy) may contribute to mucosal changes [20] visible during bronchoscopy. The high OR underscores the importance of vigilant inspection of the upper airway in patients with suspected or confirmed malignancies, as early identification of laryngopharyngeal involvement may influence staging, treatment planning, and prognosis [21].

It should also be emphasized that diseases involving the oral cavity and oropharynx are generally easier to diagnose because they are often accompanied by specific clinical symptoms (e.g., hoarseness, dysphagia) and can be directly examined without specialized equipment. In contrast, laryngeal and vocal cord lesions may remain under-recognized until they are incidentally identified during bronchoscopy, particularly in patients with coexisting pulmonary disease. Of the 21 laryngeal abnormalities detected, 18 were located in the supraglottic region, whereas only 3 were identified in the subglottic region. Subglottic lesions accounted for a minority of cases and were less readily apparent on bronchoscopy. This distribution highlights the relative diagnostic challenge of subglottic lesions, which tend to be under-detected compared with supraglottic abnormalities. Their subtle mucosal changes and hidden anatomical location in the early stages may explain the lower detection rate, underscoring the complementary role of laryngoscopy in comprehensive evaluation of the upper airway. These observations highlight the complementary rather than competitive roles of bronchoscopy and laryngoscopy in comprehensive airway evaluation and underscore the need for a systematic approach that integrates both procedures when feasible.

A younger age (<74 years) and male sex were independent predictors of abnormal findings. The mechanisms underlying these associations remain unclear; however, several hypotheses have been proposed. Younger patients may be more likely to present for evaluation at earlier stages of the disease, when subtle mucosal abnormalities remain detectable. Alternatively, certain behavioral or environmental exposures, such as smoking, alcohol consumption, or occupational irritants, may differ between age groups and sexes, influencing the prevalence of upper airway pathology [22,23,24]. The higher likelihood of abnormalities in male patients is consistent with epidemiological data showing an increased prevalence of laryngeal and pharyngeal cancers among men globally [25,26]. These findings suggest that clinicians should maintain a high index of suspicion for upper airway involvement in younger and male patients, particularly when they present with non-specific symptoms.

One of the most clinically meaningful findings of this study was the frequency at which upper airway lesions were detected incidentally during routine bronchoscopy, even in patients not initially referred for upper airway symptoms. In our cohort, 72 patients (3.2%) showed visible abnormalities in the oropharyngeal, laryngeal, and vocal cord regions during bronchoscopy. This may appear to be a relatively small proportion; however, we consider it clinically relevant given the potential implications of missed upper airway lesions, particularly in patients with malignancies. In more than half of these cases, further evaluation by otolaryngologists was pursued, allowing for more definitive diagnosis and management. This highlights the role of bronchoscopy as a diagnostic tool for lower airway disease and adjunctive screening method for upper airway abnormalities, particularly in resource-limited settings or when laryngoscopic evaluation is delayed.

Acknowledging the limitations of the current standard diagnostic tools for upper airway assessment is important. Laryngoscopy is considered the gold standard for evaluating vocal cords and supraglottic structures [7]; however, it has limited utility in visualizing the subglottic region and proximal trachea. Similarly, cross-sectional imaging modalities such as CT and MRI are excellent for identifying deep tissue involvement or extrinsic compression; however, they may miss superficial mucosal lesions or dynamic airway changes that are readily apparent during direct endoscopic visualization [8,10]. Flexible bronchoscopy offers a unique opportunity for real-time, wide-field inspection of the upper and lower airways in a single session, allowing for a more comprehensive evaluation of respiratory symptoms that may otherwise be attributed solely to intrathoracic pathology. Bronchoscopy, unlike laryngoscopy, offers the advantage of observing not only the upper airways but also the lower airways. However, it has the disadvantage of being unable to perform biopsies on lesions requiring them in the upper airways. Therefore, the two procedures should be performed in a complementary manner. In addition, meticulous attention by the operator is required when detecting upper airway lesions with bronchoscopy. If an abnormal finding is observed, it is preferable to perform confirmation and biopsy using laryngoscopy. However, if this cannot be achieved due to institutional or patient-related circumstances, it is advisable to verify the finding through imaging studies such as CT or MRI.

Despite the strengths of this study, including its relatively large sample size, use of real-world data, and standardized procedural criteria, it has some limitations. First, because this study was retrospective and observational, an inherent risk of selection and information biases was observed. Not all patients with observed abnormalities underwent confirmatory laryngoscopic evaluation, either because of logistical constraints or patient refusal, which may have resulted in under-reporting or misclassification. However, this was due to the emphasis on patients’ autonomy in diagnosis and treatment in domestic medical guidelines, as well as the restrictions imposed by the national health insurance system regarding the cost of additional examinations, which made it impossible to perform further evaluations, including laryngoscopy, for all patients. Second, the relatively small number of procedures performed by inexperienced operators limits their ability to detect subtle differences in diagnostic performance. Third, operator experience was defined solely by procedural volume, without accounting for other important factors, such as training background, supervision quality, or ongoing procedural competency assessments. Finally, we did not assess inter-observer variability in abnormality detection, which could have influenced the generalizability of the results.

## 5. Conclusions

This study demonstrated that flexible bronchoscopy has an underappreciated value in detecting upper airway abnormalities, particularly in patients with malignancy, and that abnormal findings can be reliably identified regardless of operator experience. Our findings support routine inspection of the upper airway during bronchoscopy, especially in male patients, younger individuals, and those with known or suspected malignant diseases. Future prospective studies incorporating routine laryngoscopic confirmation, standardized upper airway screening protocols, and long-term outcome data are warranted to further validate and refine the role of bronchoscopy in upper airway assessment. The integration of upper airway evaluation into bronchoscopic practice may ultimately improve early detection of clinically significant lesions and contribute to more comprehensive patient care.

## Figures and Tables

**Table 1:** Baseline characteristics of study subjects.

	Overall Group (*n* = 2229)
Age, years	65.4 ± 13.4
Male gender	1413 (63.4)
Operator	
Experienced	2092 (93.9)
Inexperienced	137 (6.1)
Presence of malignancy	
Benign disease	1360 (61.0)
Malignant disease	869 (39.0)
Additional consultation	
No consultation	32 (1.4)
Consultation	40 (1.8)
Not applicable	2157 (96.8)

The data are presented as mean No (%).

**Table 2:** Detection of abnormalities by experienced vs. inexperienced operators in anatomical regions.

		Operator		*p*-Value
		Inexperienced (*n* = 137)	Experienced (*n* = 2092)	
Oral cavity	Normal findings	137 (100)	2091 (100)	1.000
	Abnormal findings	0 (0)	1 (0)	
Larynx	Normal findings	137 (100)	2071 (99)	0.636
	Abnormal findings	0 (0)	21 (1)	
Vocal cords	Normal findings	132 (96)	2045 (98)	0.248
	Abnormal findings	5 (4)	47 (2)	
Total	Normal findings	132 (96)	2024 (97)	0.802
	Abnormal findings	5 (4)	68 (3)	

The data are presented as mean No (%).

**Table 3:** Detection of oral and laryngeal region abnormalities by presence of malignancy.

		Presence of Malignancy		*p*-Value
		Benign (*n* = 1360)	Malignant (*n* = 869)	
Oral cavity	Normal findings	1360 (100)	868 (100)	0.390
	Abnormal findings	0 (0)	1 (0)	
Larynx	Normal findings	1354 (100)	854 (98)	0.002
	Abnormal findings	6 (0)	15 (2)	
Vocal cords	Normal findings	1338 (98)	839 (96)	0.005
	Abnormal findings	22 (2)	30 (4)	
Total	Normal findings	1334 (98)	822 (95)	0.000
	Abnormal findings	26 (2)	47 (5)	

The data are presented as mean No (%).

**Table 4:** Multivariate analysis of abnormal bronchoscopic finding group.

Variables	Category	Abnormality		Univariate Analysis	
		None (*n* = 2156)	Presence (*n* = 73)	OR (95% CI)	*p*-Value
Sex	Female	802 (98)	14 (2)	Reference	
	Male	1354 (96)	59 (4)	2.069 (1.138–3.761)	0.017
Age	≥74	560 (98)	11 (2)	Reference	
	<74	1596 (96)	62 (4)	2.404 (1.247–4.634)	0.009
Operator	Inexperienced	132 (96)	5 (4)	Reference	
	Experienced	2024 (97)	68 (3)	0.648 (0.251–1.672)	0.370
Presence of malignancy	Benign disease	1334 (98)	26 (2)	Reference	
	Malignant disease	822 (95)	47 (5)	3.030 (1.829–5.017)	0.000

OR, odds ratio; CI, confidence interval. The data are presented as mean No (%).

## Data Availability

The data presented in this study are available on request from the corresponding author.

## Abbreviations

The following abbreviations are used in this manuscript:

CT	Computed tomography
MRI	Magnetic resonance imaging
OR	Odds ratio
OL	Otolaryngology
CI	Confidence interval
SPSS	Statistical Package for the Social Sciences
